# Polymer Composite Films with P(VDF-TrFE) and Molecular Ferroelectric Tris(hydroxymethyl) Nitromethane: Improvement of Their Ferroelectric Properties

**DOI:** 10.3390/polym17030354

**Published:** 2025-01-28

**Authors:** Marianela Escobar-Castillo, Samet Duman, Doru C. Lupascu

**Affiliations:** Institute for Materials Science and Center for Nanointegration Duisburg-Essen (CENIDE), University of Duisburg-Essen, 45141 Essen, Germany; sametduman10@hotmail.de (S.D.); doru.lupascu@uni-due.de (D.C.L.)

**Keywords:** P(VDF-TrFE), molecular ferroelectrics, polymer-composite, polarization, THNM

## Abstract

Polymer composites of P(VDF-TrFE) and Tris(hydroxymethyl) nitromethane as filler material with different concentrations have been prepared. Tris(hydroxymethyl) nitromethane is an organic ferroelectric material with low preparation cost and easy processing, and it is also lightweight. Its properties enable it to be a potential candidate for use as filler material in polymers to improve their ferroelectric, dielectric, and piezoelectric properties. We investigated the effect of filler content on the ferroelectric and dielectric properties of the polymer. Our results show that Tris(hydroxymethyl) nitromethane retains its crystallinity after embedding it in the polymer matrix. It does not alter the crystalline ferroelectric β-phase of the polymer. All composites possess higher polarization compared to pure P(VDF-TrFE). Up to 11.4 µC/cm^2^ remnant polarization and a dielectric constant of 14 at 1000 Hz have been obtained with the free-standing 10 wt% composite film.

## 1. Introduction

Polymer composites can fill the application gap where ceramics and other materials do not fulfil the device requirements. They possess advantages like flexibility, toughness, light weight, and high dielectric breakdown strength. Polymer composites enable the utilization of the combined advantages of the polymer matrix and the filler material and are used in different fields. Among the polymers for energy applications, P(VDF-TrFE) is one of the most investigated ones due to its chemical stability, biocompatibility, ferroelectric nature, large spontaneous polarization, and low leakage current. Ferroelectric β-phase P(VDF-TrFE) is a semi-crystalline material with an amorphous matrix where the fluorine atoms form polar point groups in the molecule, which is the basis requirement for ferroelectricity. It finds applications in energy harvesters, sensors, batteries, non-volatile random-access memories, transistors, and tissue engineering, among others [1,2,3]. One way to improve its chemical or physical properties is the preparation of polymer composites. Intensive research has been carried out by preparing P(VDF-TrFE) composites with functional fillers. The used filler materials are mostly inorganic particles like BaTiO_3_, PZT (lead zirconate titanate), and others [2,4]. The preparation of such ceramic particles includes high-temperature calcination steps [5,6], which are controversial in the global demand for CO_2_ reduction. In this sense, using organic single-component ferroelectrics (molecular ferroelectrics) as filler material becomes a potential alternative. Organic single-component materials are attracting more research interest due to their easy processability, low-cost preparation, biocompatibility, homo-chirality, and tunable chemical structure. In recent years, many organic single-component ferroelectric materials with excellent properties have been reported [7,8,9,10], namely pure organic compounds and hybrid organic–inorganic compounds. Few contributions of polymer composites with molecular ferroelectrics can be found in the literature. Recently, researchers prepared P(VDF-TrFE)/MDABCO-NH_4_I_3_ composites and found an increase in the relative permittivity and remnant polarization compared to the pure polymer [11]. Furthermore, Baptista et al. fabricated different MDABCO-NH_4_I_3_/polymer composites as potential flexible energy harvesters [12]. Ai et al. reported the properties of an interesting molecular ferroelectric, namely 2-(Hydroxymethyl)-2-nitro-1,3-propanediol (Tris(hydroxymethyl) nitromethane, THNM) [13] with a Curie temperature of around 362 K. The molecules of this compound are loosely connected to each other by hydrogen bonds formed between their hydroxyl groups. Its ferroelectricity originates from the alignment of C-N bonds in the crystal structure. It possesses a six-fold vertex domain structure, good piezoelectric properties, and multiple crystallographic equivalent polarization directions. Materials with several polarization directions are strong candidates for thin film applications and filler material in polymer composites. Combining THNM and P(VDF-TrFE), new metal-free composites with improved ferroelectric, dielectric, and piezoelectric properties can be prepared.

The present study uses the solution casting method to prepare flexible organic ferroelectric composite films based on THNM with P(VDF-TrFE) as a polymer matrix. We investigated the film morphology, crystallinity, and the ferroelectric and dielectric properties.

## 2. Materials and Methods

Commercial tris(hydroxymethyl) nitromethane (THNM, Sigma-Aldrich, Chemie GmbH, Taufkirschen, Germany, 98% purity) was recrystallized in methanol before its use as polymer filler. Free-standing composite films were prepared by mixing P(VDF-TrFE), (Piezotec FC30, Pierre-Bénite, France) and THNM (previously dissolved separately in a DMSO/acetone mixture) at room temperature. The mixture was stirred briefly and cast onto a glass substrate. The glass substrate was heated at 60 °C for 4 h on a hot plate. Then, it was heated further and kept at 100 °C for 8 h in a vacuum oven at 100 mbar. After natural cooling, the polymer film was carefully removed from the glass substrate. Silver electrodes were sputtered onto both sides of the films (Cressington Scientific Instruments, 208 HR Turbo-Sputter Coater, Watford WD 19 4BX, UK). A Solartron 1260 Impedance Analyzer (Solartron Analytical, Farnborough Hampshire, UK) was used for dielectric measurements, and aixACCT Systems (TF Analyzer 3000, Aachen Germany) were used for polarization measurements (bipolar triangular waveform). An electron microscope (Philips XL 30 ESEM TMP, OR, USA) and a digital microscope (Keyence VHX-7000, Neu-Isenburg, Germany) were used for film morphology analysis and determination of the film thicknesses. Material phase analysis was performed by XRD using a Panalytical Empyrean diffractometer, Cu Kα radiation, Almelo, The Netherlands). A Linseis Chip-DSC 100 calorimeter (Linseis Messgeraete GmbH, Selb, Germany) was used for DSC analysis. For these measurements, 10 mg film was cut into small pieces and placed in a DSC aluminum crucible. The DSC of pure THNM was performed using the recrystallized commercial powder. The heating rate for all DSC measurements was 10 °C/min.

## 3. Results and Discussion

### 3.1. Analytical Characterization

**Figure 1:**
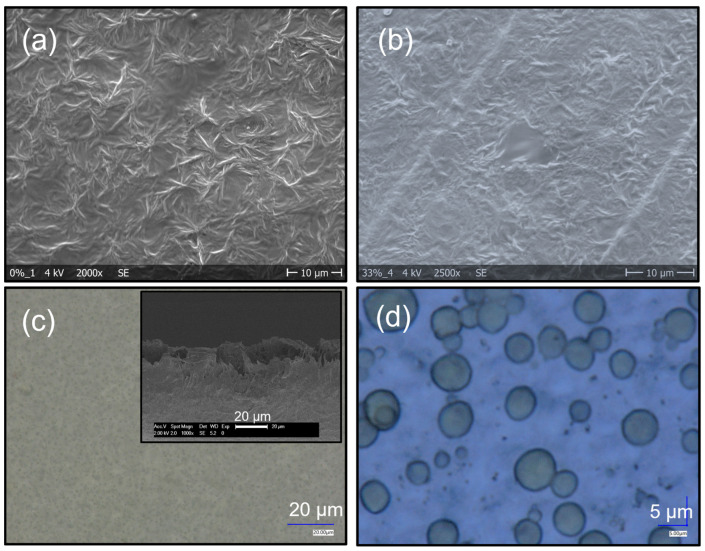
SEM (**a**,**b**) and digital microscope images (**c**,**d**). P(VDF-TrFE) (**a**) and 10 wt% composites (**b**–**d**). Digital images were taken in transmission mode.

**Figure 2:**
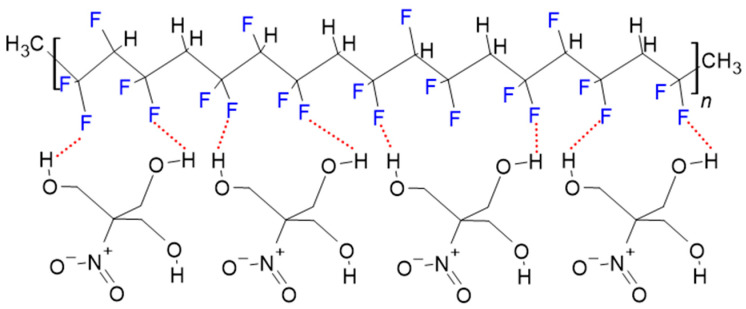
Hydrogen bond interaction between the polymer’s fluorine atoms and the −OH groups of THNM allows a smooth transition from one phase to the other.

The morphology of the films was investigated using a scanning electron microscope (SEM) and a digital microscope in transmission mode. The images are shown in Figure 1. The polymer films show characteristics of dendrite-like structures on the surface (Figure 1a,b) that are typical of this polymer [4,14]. Transmission images of the composites (Figure 1c,d) show that the THNM filler is homogeneously distributed in the polymer matrix. These dots are different in size, from 8 to <1 µm diameter, and have a low degree of connection area between them. Due to the presence of fluorine atoms and hydroxyl groups in the polymer and the filler material, hydrogen bonds between these groups are formed (−F∙∙∙∙HO−). This strong interaction between the THNM and the polymer matrix guarantees interface compatibility (Figure 2). This characteristic is advantageous when compared with inorganic filler materials with no interface compatibility with the polymers. From the microscope images in Figure 1, we can see no macroscopic cracks on the surface of the films. The absence of cracks and other morphological defects is advantageous for the sample stability under high electric fields. The films are also flexible and have an average thickness of around 30–40 µm, which has been determined using SEM and optical microscopes (Figure 1c insert).

**Figure 3:**
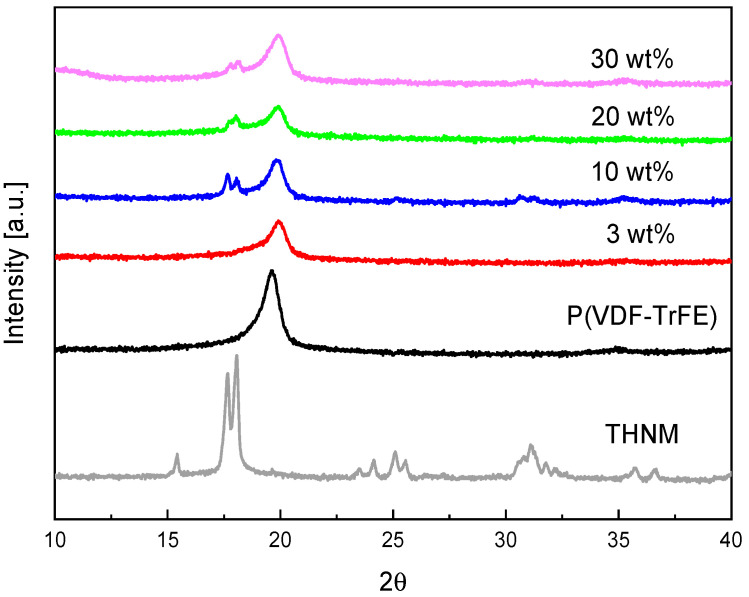
XRD diffractograms of the polymer composites containing different wt% of THNM.

The crystallinity of the films was analyzed by XRD measurements (Figure 3). XRD-signals of THNM are intense, showing high crystallinity. It crystallizes in the triclinic system with the polar space group P1 and is not centrosymmetric [13]. Meanwhile, P(VDF-TrFE) possesses one main peak at around 19.5°, characteristic of the ferroelectric β–phase [14]. The composite diffractograms clearly show the main peaks of the P(VDF-TrFE) and the THNM filler at around 19° and 17.5°, respectively, which means that the film-preparation process does not affect the chemical stability and crystalline nature of both materials. This is an important fact since the THNM, whose molecules are connected only by hydrogen bonds, was dissolved together with the polymer in an organic solvent during the film-preparation process, and it recrystallizes after evaporation of the solvent.

**Figure 4:**
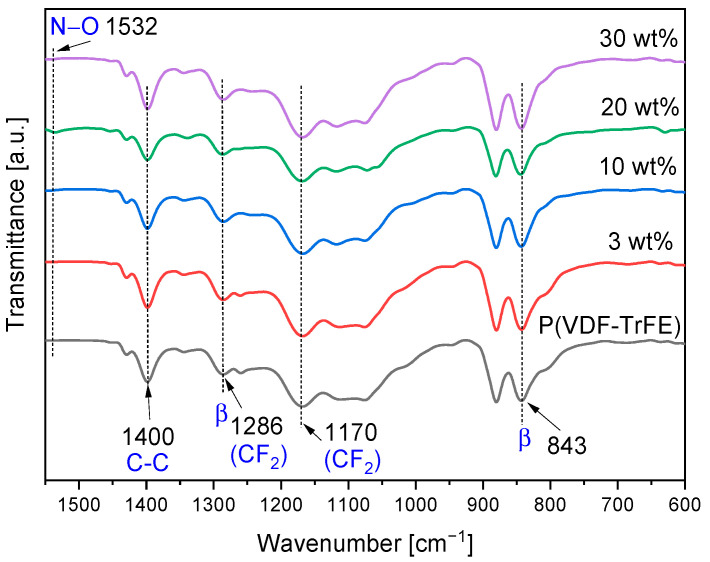
FTIR spectra of the composites containing different wt% of THNM.

The co polymer P(VDF-TrFE) is formed by VDF and TrFE units. The TrFE units stabilize the ferroelectric β-phase in the polymer and enhance its crystallinity [1]. Such polymers possess ferroelectric behavior without any post-polymerization treatment. In comparison, pure PVDF, which crystallizes in the thermally stable α-phase, needs stretching or hot-pressing steps to transform it into the β-phase [14]. The β-phase content in P(VDF-TrFE) depends on the amount of TrFE units, the processing method, temperature, solvent, and filler material. Since the electrical properties of P(VDF-TrFE) depend on the degree of crystallinity, phase content [3], crystallite size, and morphology, adding functional fillers can significantly influence these characteristics. For pure PVDF, the addition of nanofillers in the polymer matrix can lead to the formation of the β-phase [14]. Filler inclusion in the P(VDF-TrFE) matrix to enhance the β-phase formation and increase crystallinity has been the focus of many research works [3,14,15]. FTIR measurements were performed to analyze the effect of THNM on the polymer β-phase (Figure 4). Comparing the spectra of the films with different THNM content, little differences can be seen between them. Characteristic peaks for the ferroelectric β-phase of the P(VDF-TrFE) are 843 and 1286 cm^−1^. Both signals are associated with trans-isomer sequences longer than TTTT and TTT units, respectively [16,17]. The transmission peak at 1286 cm^−1^ corresponds to the symmetric stretching vibration of CF_2_ [18]. The peak at around 1170 cm^−1^ is characteristic of the antisymmetric stretching and rocking vibration of CF_2_ units [18]. The strong peak at 1400 cm^−1^ is related to the –CH_2_ wagging frequency [18]. These peaks are present in all spectra, confirming that the ferroelectric β-phase in the polymer remains after adding THNM. The characteristic peak at 1532 cm^−1^ corresponds to the N–O stretching vibration of the nitro group in THNM [19], which is absent in pure P(VDF-TrFE). In conclusion, the FTIR results show that the THNM filler does not significantly affect the β-phase content of the P(VDF-TrFE).

**Figure 5:**
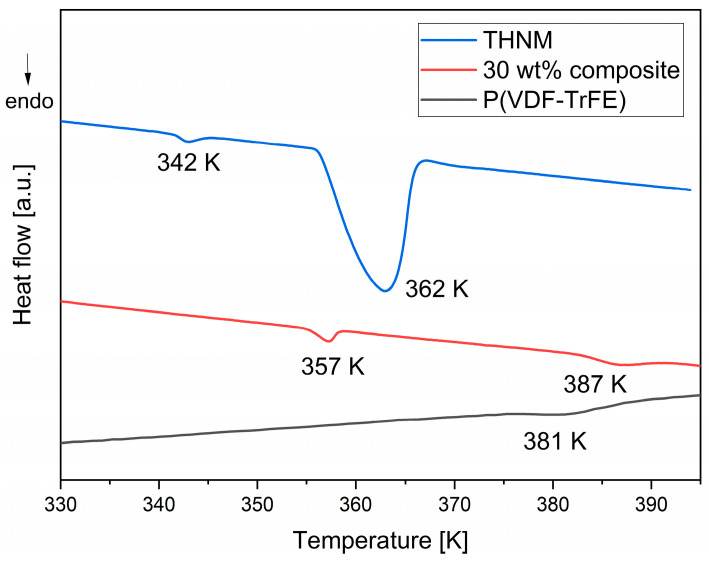
DSC analysis of the material phase transitions.

Ferroelectric materials are characterized by a Curie temperature, where a transition from the ferroelectric to the paraelectric phase occurs. This transition is associated with heat transformation in the material during this process. Phase transitions are usually measured using thermal analysis techniques, such as use of a DSC calorimeter. DSC measurements of the films were performed to check the ferroelectric–paraelectric phase transition of THNM, P(VDF-TrFE), as well as the composites. Our DSC analysis shows that pure THNM crystalline powder possesses two structural phase transitions at 342 K and 362 K; this result agrees well with the results obtained by Ai et al. [13] (Figure 5). The authors demonstrated that THNM shows an ordered structural state below 342 K, a slowly rotating state between 342 K and 362 K, and a highly disordered state at temperatures higher than 362 K. The ferroelectric–paraelectric phase transition is due to the change in the degree of order–disorder of the THNM molecules. At a temperature higher than 362 K, the molecules freely rotate, yielding a highly symmetric structure with a face-centered cubic lattice. No polarization in any direction can be detected above this temperature. On the other hand, the co-polymer P(VDF-TrFE) with VDF/TrFE ratio of 70/30 has one phase transition at around 381 K (Figure 5); this endothermic peak corresponds to its Curie temperature according to the material properties specification from the company Piezotech, which commercializes the used polymer powder. The DSC curve of the 30 wt% composite film clearly shows two characteristic peaks: at 357 K, corresponding to THNM, and at 387 K, which can be attributed to the polymer phase transition. The presence of both DSC peaks in the composite film further indicates that the organic filler material and P(VDF-TrFE) retain their chemical characteristics and do not lose their ferroelectric properties when preparing the composites.

### 3.2. Electrical Properties and Polarization

The addition of functional fillers to a polymer matrix influences its dielectric permittivity. Several factors can influence it, such as the effective molecular structure of the polymer, its chemical or physical interaction with the filler material, the distribution of the fillers in the polymer matrix, the existence of particle filler aggregates, the amount of filler material, and the applied field. The effective dielectric constant (*ɛ_eff_*) of a composite material can be calculated by effective medium approximation methods [20,21]. The Maxwell–Garnett approximation (Equation (1)) or the Lichtenecker (Equation (2)) model can be used for composites where the filler consists of homogeneous spherical particles distributed in a matrix [22]. In these equations, *ɛ_T_* is the dielectric constant of THNM, *ɛ_P_* is the dielectric constant of the polymer, and *V_T_* is the volume fraction of THNM. From the equations, one can see that filler materials with a high dielectric constant can enhance the effective dielectric constant of the composite. P(VDF-TrFE) and the THNM filler material have a dielectric constant of approximately 10 [2] and 6 [13], respectively. From this, it is expected that THNM will not enhance the effective dielectric constant of the composite in the concentration range we used.

(1) $$\varepsilon_{eff}=\varepsilon_{P}\left[\frac{\varepsilon_{T}+2\varepsilon_{P}+2V_{T}\left(\varepsilon_{T}-\varepsilon_{P}\right)}{\varepsilon_{T}+2\varepsilon_{P}-V_{T}\left(\varepsilon_{T}-\varepsilon_{P}\right)}\right]$$

(2) $$ln\varepsilon_{eff}=V_{T}ln\varepsilon_{T}+(1-V_{T})ln\varepsilon_{P}$$

The composite system possesses different types of polarization. For multicomponent polymers, the Maxwell–Wagner relaxation generates interfacial polarization [20]. P(VDF-TrFE) and THNM are polar materials whose permanent dipoles rotate by applying an electric field, generating dipole polarization. Additionally, all polymers show electron- and atomic (vibrational) polarization [20]. The dielectric constant at frequencies lower than 10 Hz shows interfacial polarization mechanisms, while at frequencies below 10^9^ Hz, dipole polarization processes involve the movement of molecules. 

**Figure 6:**
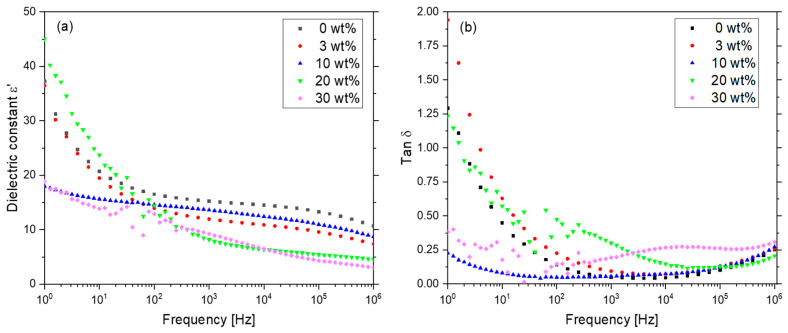
Frequency dependencies of the dielectric constant (**a**) and dielectric loss (**b**) of the composites at room temperature.

Figure 6a summarizes the experimental results of the frequency dependence of the dielectric constant (ε’) at room temperature. The real part of the dielectric constant of the film ε’ decreases with increasing frequency, characteristic of composites with dipole and interfacial polarization [20] and for a dipolar relaxation of the -CF_2_-CH_2_- groups in the polymer [23]. Adding THNM as filler material lowers the ε’ of the composites in comparison to pure P(VDF-TrFE); this effect can be attributed to the influence of the THNM filler, which has a lower ε’ than the polymer [13]. The smooth values change under 10 Hz for the 10 wt% and 30 wt% composites, which indicates that the interfacial polarization effect between filler and polymer matrix is low [23] due to the low ε’ difference between the filler and the polymer [20] and the hydrogen bonds between the polymer’s fluorine atoms and the filler material’s hydroxyl groups (−F∙∙∙∙HO−). These hydrogen bonds avoid an abrupt change at the polymer–filler interface. However, for the other samples (0 wt%, 3 wt%, 20 wt%), the ε’ values change more significantly in this frequency range, probably originating from the interface effect between composite–electrode.

We compared the experimental ε’ values with the predictions obtained using the Maxwell–Garnett approximation (Equation (1)) for a frequency of 10^3^ Hz. The value of *ɛ_T_* is 6 and was taken from the research work of Ai et al. [13]. The used value of *ɛ_P_* was 10 [2]. The calculated results (Table 1) show that the higher the volume fraction of THNM, the lower the expected *ɛ_eff_* of the composites. Some deviations in the obtained experimental values can be seen. This is due to several factors, like the non-uniform distribution of the filler, that cause an inhomogeneous electric field distribution in the polymer matrix, the interface effect between the film and the silver electrode, etc.

The dielectric loss (tan δ) in the composites under 10 Hz follows a similar trend to the ε’ values (Figure 6b). In the range of 10 to 10^5^ Hz, tan δ of 20 wt% and 30 wt% follow a different trend; this could be attributed to the formation of conduction pathways along the filler material in the composites, especially at filler concentrations of 30 wt%, which enhances the DC conductivity, as well as the dielectric loss. Comparing the tan δ values of all composites, the sample with 10 wt% filler content shows a small value of tan δ, which is relatively constant in the whole frequency range. Low tan δ values can result from good surface compatibility between THNM and P(VDF-TrFE). The formed hydrogen bonds (Figure 2) at the molecular level avoid an abrupt change from one material to another, leading to a more homogeneous microstructure in the composites. The slight difference in dielectric constants between both materials also enables a homogeneous distribution of the applied electric field in the composite films, thus reducing charge accumulation at the filler–polymer interfaces. Such processes can cause energy dissipation in the form of heat and enhance the dielectric loss.

**Figure 7:**
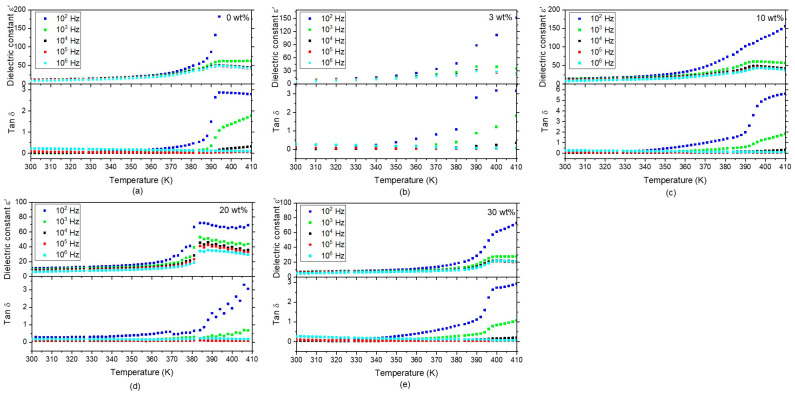
Temperature dependencies of the dielectric constant ε’and tan δ for the P(VDF-TrFE) (**a**) and the composite films (**b**–**e**). All samples show enhanced ε’ values at the polymer phase transition temperature. Tan δ values of all samples are stable at frequencies 10^4^–10^6^ Hz.

The temperature dependence of ε’ can be seen in Figure 7. We observe a steady relative permittivity in all films in the frequency range of 10^3^ to 10^6^ Hz and temperature range of 300 K to 370 K. At 10^2^ Hz; the conduction process is more pronounced and increases ε’ at higher temperatures. All films’ peak maxima at around 390 K can be assigned to the polymer ferroelectric–paraelectric phase transition. Furthermore, in the temperature dependence of tan δ graphs, steady values can be seen at frequencies higher than 10^3^ Hz for all films. Only at higher temperatures do the tan δ values increase at the temperature range of the polymer phase transition.

**Figure 8:**
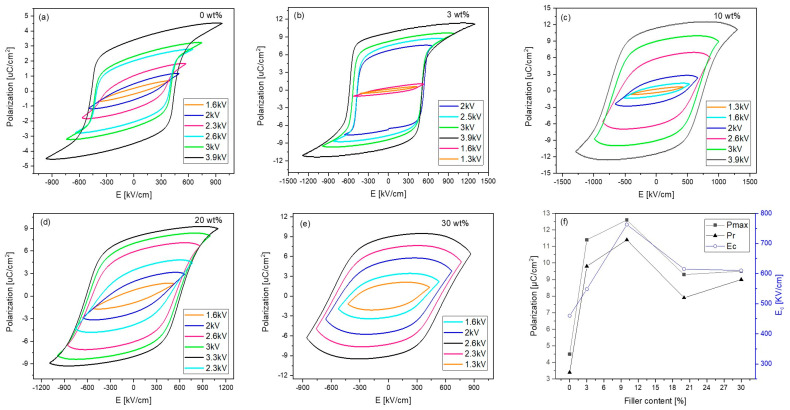
Polarization hysteresis loops of the composites with different filler content measured at 1 Hz at room temperature. (**a**–**c**) show the electric field stability of the samples until an applied voltage of 3.9 kV. (**d**,**e**) composites with 20 wt% and 30 wt% were unstable by applied voltages higher than 3.3 kV and 2.6 kV respectively. (**f**) show the comparison of P_max_, P_r_, E_c_ between the prepared composites with the pure P(VDF-TrFE).

**Figure 9:**
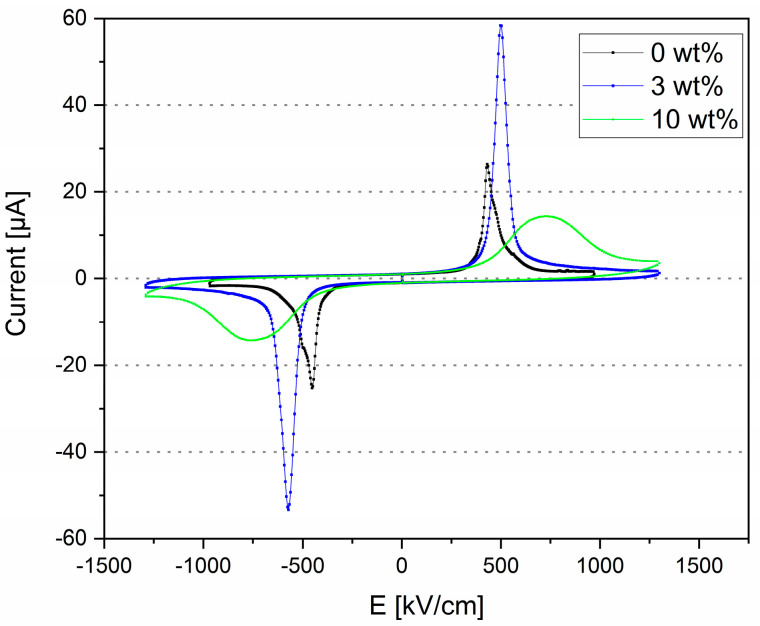
Room temperature, current density curves of the films at 1 Hz.

Typical room temperature P-E hysteresis loops were obtained for all films (Figure 8), displaying the ferroelectric nature of the films. The addition of THNM filler significantly enhances the maximal polarization (P_max_) of all composites (Figure 8f), with the 10 wt% composite having the highest Pmax and the highest remnant polarization (P_r_) of 12.6 µC/cm^2^ and 11.4 µC/cm^2^, respectively. Comparing the polarization loops (Figure 8a–e), it is clear that the ferroelectric domain switching in the pure polymer needs approximately 570 kV/cm (Figure 8a, the loop at 2.6 kV), which is higher than for the 3 wt% (537 kV/cm, in Figure 8b the loop at 2 kV) and 10 wt% (526 kV/cm, in Figure 8c the loop at 2.6 kV) composites. In the literature, several authors reported much higher fields than 700 kV/cm for the start of domain switching in pure P(VDF-TrFE) [3,24], so we can conclude that adding the THNM filler lowers the necessary field to switch the ferroelectric domains in the P(VDF-TrFE) matrix. The corresponding I-E loop in the pure polymer (Figure 9) follows a simple reversal response. It originates from the cooperative switching of the CF_2−_ and CF_3_ units in the P(VDF-TrFE) chain [25]. The I-E loop of the 3 wt% composite follows a similar response, but the leakage current increases for the other composites (10 wt%, 20 wt%, 30 wt%). In comparison, the 3 wt% and 10 wt% composites were stable under applying a field of up to 1300 kV/cm, whereas the 20 wt% and 30 wt% composites break down using fields higher than 1100 kV/cm and 870 kV/cm, respectively. Leakage current in the composites with high filler content can stem from forming ionizable hydroxyl groups that can create charge–conduction pathways. However, the 3 wt% composite showing well-saturated hysteresis loops with P_max_ of 11.4 µC/cm^2^, Pr of 9.8 µC/cm^2^, 548 kV/cm coercive field, and low leakage current is the sample with the best results in our study in the sense of ferroelectricity.

**Figure 10:**
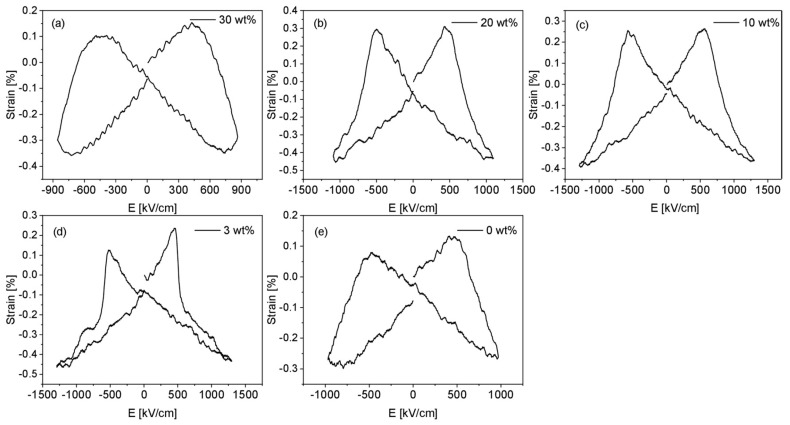
Strain–field hysteresis loops display the electrostrictive response of the material at room temperature. All composite films (**a**–**d**) and pure P(VDF-TrFE) (**e**) show reverse butterfly hysteresis loops characteristic for a typical piezoelectric response.

All films show a reverse butterfly bipolar shape (Figure 10), typical of piezoelectric materials with a negative piezoelectric coefficient d_33_. From the loops, one can see that the strain value of the composite tends to be higher than the pure P(VDF-TrFE). Since strain is correlated to the piezoelectric coefficient d_33_, its improvement could also be interesting for flexible piezoelectric devices. However, this behavior will be investigated in detail in future work.

## 4. Conclusions

Free-standing, flexible P(VDF-TrFE) composite films with metal-free molecular ferroelectric THNM as filler material were prepared using the solution casting method. Analysis of their dielectric and ferroelectric properties was performed. The polymer composites with approximately 30–40 µm thickness show mechanical stability, and their crystalline nature was confirmed by XRD, DSC, and FTIR analysis. All composites show improved ferroelectric properties compared to pure P(VDF-TrFE). The 3 wt% composite with the lowest leakage current had a P_max_ of 11.4 µC/cm^2^ and a P_r_ of 9.8 µC/cm^2^ at an applied electric field of 1300 kV/cm. By preparing these polymer composites, we have demonstrated that metal-free organic ferroelectric materials can be an alternative to inorganic ceramic fillers to improve the ferroelectric properties of the P(VDF-TrFE). The breakdown strength of the composite films is high due to the polymer–THNM compatibility caused by the hydrogen bond interactions between them. Due to these characteristics and their easy processability, the prepared composite films can have potential applications in flexible energy harvesting devices, not only due to their ferroelectricity, but also due to the ability of polymer films to have stability for high mechanical strain without risk of breaking.

## Figures and Tables

**Table 1:** Comparison of the dielectric constant ε’ of the (PVDF-TrFE)/THNM composites for a frequency of 10^3^ Hz.

Dielectric Constant	3 wt%	10 wt%	20 wt%	30 wt%
Maxwell–Garnett model	9.7	9.0	8.2	7.5
Experimental values	11.9	13.6	8.2	9.2

## Data Availability

The original contributions presented in this study are included in the article. Further inquiries can be directed to the corresponding author.

## Abbreviations

The following abbreviations are used in this manuscript:

P(VDF-TrFE)	Poli(vinylidene fluoride-co-trifluoroethylene)
THNM	Tris(hydroxymethyl) nitromethane

## References

[1] Qian X., Xin C., Lei Z., Zhang Q.M. (2023). Fluoropolymer ferroelectrics: Multifunctional platform for polar-structured energy conversion. Science.

[2] Priya L., Swain B., Rajput S., Parida S. (2023). Advances in P(VDF-TrFE) Composites: A Methodical Review on Enhanced Properties and Emerging Electronics Applications. Condens. Matter.

[3] Abdolmaleki H., Haugen A.B., Buhl K.B., Daasbjerg K., Agarwala S. (2023). Interfacial Engineering of PVDF-TrFE toward Higher Piezoelectric, Ferroelectric, and Dielectric Performance for Sensing and Energy Harvesting Applications. Adv. Sci..

[4] Shanshan G., Escobar-Castillo M., Shvartsman V.V., Karabasov M., Lupascu D.C. (2019). Electrocaloric Effect in P(VDF-TrFE)/Barium Zirconium Titanate Composites.

[5] Zhao X., Liu W., Chen W., Li S. (2015). Preparation and properties of BaTiO_3_ ceramics from the fine ceramic powder. Ceram. Int..

[6] Xu Z., Chu R., Li G.S., Yin Q. (2004). Preparation of PZT powders and ceramics via a hybrid method of sol–gel and ultrasonic atomization. Mater. Sci. Eng. B.

[7] Zhang T., Xu K., Li J., He L., Fu D.W., Ye Q., Xiong R.G. (2022). Ferroelectric hybrid organic–inorganic perovskites and their structural and functional diversity. Natl. Sci. Rev..

[8] Bergentti I. (2022). Recent advances in molecular ferroelectrics. J. Phys. D Appl. Phys..

[9] Zhang Z.X., Ni H.F., Tang J.S., Huang P.Z., Luo J.Q., Zhang F.W., Lin J.H., Jia Q.Q., Teri G., Wang C.F. (2024). Metal-Free Perovskite Ferroelectrics with the Most Equivalent Polarization Axes. J. Am. Chem. Soc..

[10] Liu H.Y., Zhang H.Y., Chen X.G., Xiong R.G. (2020). Molecular Design Principles for Ferroelectrics: Ferroelectrochemistry. J. Am. Chem. Soc..

[11] Li C., Cai Y., Xie Y., Sheng C., Qin Y., Cong C., Qiu Z.J., Liu R., Hu L. (2023). Enhanced dielectric/ferroelectric properties of P(VDF-TrFE) composite films with organic perovskite ferroelectrics. Appl. Phys. Express.

[12] Baptista R., Moreira G., Silva B., Oliveira J., Almeida B., Castro C., Rodrigues P., Machado A., Belsley M., de Matos Gomes E. (2022). Lead-Free MDABCO-NH_4_I_3_ Perovskite Crystals Embedded in Electrospun Nanofibers. Materials.

[13] Ai Y., Zeng Y.L., He W.H., Huang X.Q., Tang Y.Y. (2020). Six-Fold Vertices in a Single-Component Organic Ferroelectric with Most Equivalent Polarization Directions. J. Am. Chem. Soc..

[14] Wu L., Jin Z., Liu Y., Ning H., Liu X., Alamusi, Hu N. (2022). Recent advances in the preparation of PVDF-based piezoelectric materials. Nanotechnol. Rev..

[15] Chen J.X., Li J.W., Cheng C.C., Chiu C.W. (2022). Piezoelectric Property Enhancement of PZT/Poly(vinylidenefluoride-co-trifluoroethylene) Hybrid Films for Flexible Piezoelectric Energy Harvesters. ACS Omega.

[16] Ryu J., No K., Kim Y., Park E., Hong S. (2016). Synthesis and Application of Ferroelectric Poly(Vinylidene Fluoride-co-Trifluoroethylene) Films using Electrophoretic Deposition. Sci. Rep..

[17] Arrigoni A., Brambilla L., Bertarelli C., Serra G., Tommasini M., Castiglioni C. (2020). P(VDF-TrFE) nanofibers: Structure of the ferroelectric and paraelectric phases through IR and Raman spectroscopies. RSC Adv..

[18] Liew W.H., Mirshekarloo M.S., Chen S., Yao K., Tay F.E.H. (2015). Nanoconfinement induced crystal orientation and large piezoelectric coefficient in vertically aligned P(VDF-TrFE) nanotube array. Sci. Rep..

[19] Becker H., Berger W., Domschke G., Fanghänel E., Faust J., Fischer M., Gentz F., Gewals K., Gluch R., Mayer R. (1977). Organikum.

[20] Wang L., Yang J., Cheng W., Zou J., Zhao D. (2021). Progress on Polymer Composites with Low Dielectric Constant and Low Dielectric Loss for High-Frequency Signal Transmission. Front. Mater..

[21] Bărar A., Maclean S.A., Gross B.M., Mănăilă-Maximean D., Dănilă O. (2024). Mixing Rules for LeftHanded Disordered Metamaterials: Effective-Medium and Dispersion. Nanomaterials.

[22] Hambal Y., Shvartsman V., Michiels I., Zhang Q., Lupascu D. (2022). High Energy Storage Density in Nanocomposites of P(VDF-TrFE-CFE) Terpolymer and BaZr_0.2_Ti_0.8_O_3_ Nanoparticles. Materials.

[23] Mayeen A., Kala M.S., Jayalakshmy M.S., Thomas S., Philip J., Rouxel D., Bhowmik R.N., Kalarikkal N. (2019). Flexible and self-standing nickel ferrite–PVDF-TrFE cast films: Promising candidates for high-end magnetoelectric applications. Dalton Trans..

[24] Zhang X., Xia W., Ping Y., Wang R., Wei T., Xing J. (2022). Dielectric, ferroelectric, and energy conversion properties of a KNN/P(VDF-TrFE) composite film. J. Mater. Sci. Mater. Electron..

[25] Tsutsumi N., Okumachi K., Kinashi K., Sakai W. (2017). Re-evaluation of the origin of relaxor ferroelectricity in vinylidene fluoride terpolymers: An approach using switching current measurements. Sci. Rep..

